# Supplementary material for: Effect of a Health Education Intervention to Reduce Fear of Falling and Falls in Older People: A Cluster Randomized Clinical Trial Protocol
Source: Healthcare (Basel). 2024 Dec 11;12(24):2510. doi: 10.3390/healthcare12242510 (PMC11728216; doi:10.3390/healthcare12242510)
Supplement: Supplementary file 1 [file healthcare-12-02510-s001.zip › healthcare-3311150-supplementary.pdf]

**Supplementary File S1. Description of assessment tools.**

- The Barthel Index measures a person's ability to perform ten basic activities of daily living (BADLs) by providing a quantitative estimate of their degree of independence. Depending on their ability to perform each of these activities, a score of 0, 5, 10, or 15 will be assigned. The maximum score is 100, with higher scores indicating higher levels of independence. According to the literature, the Barthel Index has an inter-observer reliability (kappa index) between 0.47 and 1.00 and an intra-observer reliability (kappa index) between 0.84 and 0.97, with internal consistency ranging from 0.86 to 0.92 [44].

- The Downton Scale helps to measure risk of falling. It contains five items, adding one point for each of the categories affected [45]. It is routinely used in primary care. Scores ≥3 indicate a high risk of falling. Even though it has a low internal consistency (Cronbach's alpha = 0.51) [46], it is widely used in clinical settings.

- The Lawton-Brody scale measures instrumental activities of daily living (IADLs) by assessing people's ability to use the telephone, shop, prepare food, take care of the house, do the laundry, use means of transport, manage financial affairs, and handle their own medication. Each area scores either 1 if functional or 0 if not functional, with the maximum score being 8 [47].

- The Short Physical Performance Battery (SPPB) consists of three performance tests: balance (in three positions: feet together, semi-tandem and tandem), walking speed (over 2.4 or 4 metres) and standing up and sitting down in a chair five times. The total SPPB score is the sum of the three sub-tests and ranges from 0 to 12. Scores below 10 indicate frailty and a high risk of disability and falls. Balance, strength and speed scores as well as the final total score will be collected separately, classifying patients as frail in cases where the total score is below 10, as indicated by the Spanish Ministry of Health [48]. It has a Cronbach's alpha of 0.70 and an intraclass correlation coefficient of 0.80 [49].

- The Charlson Index measures comorbidity in older people. It is widely used to estimate the 10-year risk of death. It assesses the presence of 19 medical diagnoses (such as a history of acute myocardial infarction, cerebrovascular disease, chronic lung disease, and diabetes). The presence of three or more of these diagnoses indicates comorbidity. Its test-retest reliability, as measured using the intraclass correlation coefficient (ICC), was 0.77 [95% CI: 0.60 to 0.87] [50].

- Lobos' Mini-Examen Cognoscitivo (MEC) is the Spanish adaptation and validated version of Folstein's Mini-Mental State Examination (MMSE) [51]. The MEC is used for both dementia screening and monitoring. Its items explore five cognitive areas: Orientation, Registration, Attention and Calculation, Recall, and Language. A score below 24 indicates cognitive impairment [52]. The instrument meets the following criteria: feasibility and content, procedural, and construct validity. Test-retest reliability: weighted kappa = 0.637. MEC-30 (with cut-off point at 23/24); sensitivity = 89.8%; specificity = 75.1% (80.8% with cut-off point at 22/23); and area under the ROC curve = 0.920 [53].

- The Generalised Anxiety Disorder-7 (GAD-7) questionnaire contains seven questions that are rated between 0 and 3. Total scores range from 0 to 21, with higher scores indicating higher anxiety levels [54-56]. Scores of 5 and above obtained sensitivity values of 0.63 and specificity values of 0.9 [56].

- The Patient Health Questionnaire-8 (PHQ-8) [57] comprises eight questions that are rated between 0 and 3. Total scores range from 0 to 24, with higher scores indicating higher depression levels. Scores above 10 indicate major depression and scores above 20 suggest severe depression [58]. The sensitivity and specificity of PHQ-8 scores ≥10

for major depressive disorder (vs other + none) is 100% and 95%, respectively; and for any depressive disorder, the sensitivity and specificity is 70% and 98%, respectively [57].

- The Appraisal of Self-Care Agency Scale—Revised (ASA-R) is made up of 15 items. Total scores range from 31 to 75, with the author's recommended cut-off point being 53. Higher scores indicate greater ability to self-care. The ASA-R has proven to be a reliable (composite reliability (CR) > 0.7) and valid (average variance extracted (AVE) > 0.5) instrument for measuring self-care agency among the Spanish elderly population [59].

**Supplementary File S2. Educational programme for nurses in the intervention group**

**Table S1. Summary of the educational intervention.**

| | |
|---|---|
| **Session 1** | Presentation<br>Introduction to falls and fear of falling<br>Risk and protective factors<br>Multicomponent exercise session<br>Relaxation |
| **Session 2** | Session 1 recap<br>Falls and fear of falling recap<br>How to act in the event of a fall<br>Multicomponent exercise session<br>Relaxation |
| **Session 3** | Session 2 recap<br>Cognitive restructuring<br>Multicomponent exercise session<br>Relaxation |
| **Session 4** | Session 3 recap<br>How to act in the event of a fall<br>Cognitive restructuring<br>Coping strategies<br>Multicomponent exercise session<br>Relaxation |
| **Session 5** | Recap of previous sessions<br>Falls and fear of falling recap<br>Fall prevention<br>Coping strategies<br>Multicomponent exercise session<br>Relaxation |
| **Booster Session** | Session presentation<br>Perceptions and feelings<br>Falls and fear of falling recap<br>Risk factors<br>Fall prevention and how to act in the event of a fall<br>Coping strategies<br>Multicomponent exercise session<br>Relaxation |

Summary of the educational intervention.

**Table S2. Session 1: Presentation. Introduction to falls and fear of falling.**

| Contents | Objectives | Method | Length | Materials Required |
|---|---|---|---|---|
| **Workshop presentation** | To get acquainted with the professionals leading the intervention.<br>To get acquainted with the contents of the workshop.<br>To create a safe space.<br>o express personal expectations of the workshop. | Talk followed by a discussion<br>Reception | 15 min | Screen, computer<br>Chairs |
| **Introduction to falls and fear of falling in older people** | To be able to describe the significance of falls and the fear of falling in older people.<br>To be aware of the existing risk factors and the preventive measures available to reduce them.<br>To draw up a list of measures to reduce the risk of falls and the fear of falling (group work).<br>Homework: Drawing up a list of individual actions to reduce the risk of falls and fear of falling.<br>To help patients identify suitable short- and long-term objectives. | Brainstorming<br>Talk followed by a discussion<br>Repertory grid<br>Homework | 10 min<br>15 min<br>20 min<br>10 min | Screen, computer<br>Whiteboard, marker<br>Pen, paper |
| **Multicomponent exercise** | To demonstrate how to make a multicomponent exercise plan.<br>To help each individual develop a suitable exercise programme tailored to their needs.<br>To advise patients on the cognitive benefits of social engagement and physical activities.<br>To give positive feedback on each individual's efforts. | Demonstration and training<br>Talk followed by a discussion | 25 min | Mats |
| **Relaxation techniques** | To learn and perform relaxation techniques during the session. | Demonstration and training | 15 min | Mats |
| **Close the session** | To listen to a recap of the session.<br>To voice feelings, perceptions, and fears.<br>To remind participants of their homework. | Talk followed by a discussion | 10 min | Chairs |

Session 1 Presentation. Introduction to falls and fear of falling.

**Table S3. Session 2: Recovering from a fall.**[3]

| Contents | Objectives | Method | Length | Materials Required |
|---|---|---|---|---|
| **Recap of the first session** | To explain the important concepts taught in the previous session. | Participative lesson | 10 min | Chairs, whiteboard |
| **Setting individual objectives and measures** | To present individual measures devised to reduce the risk of falling (homework).<br>To identify suitable short- and long-term goals and measures to be taken to reduce fear of falling. | Brainstorming<br>Dialogue | 10 min<br>20 min | Screen, computer<br>Whiteboard, marker.<br>Pen, paper |
| **How to act in the event of a fall and how to handle them** | To become aware of the appropriate actions to take in the event of a fall.<br>To describe the painful emotion (anger, anxiety, hopelessness) they are feeling.<br>To list the situations or events contributing to their stress (stressors). | Talk followed by a discussion | 10 min | Screen, computer |
| | To demonstrate how to get up after a fall. | Demonstration and training | 20 min | Mats |

| | | | | |
|---|---|---|---|---|
| **Multicomponent exercise** | To demonstrate how to make a multicomponent exercise plan.<br>To give positive feedback on each individual's efforts.<br>To learn how to inform the patient of the purpose and benefits of the prescribed exercise.<br>To learn how to help patients set their own goals for a slow and steady increase in exercise.<br>Homework: doing multicomponent exercises at home. | Demonstration and training<br>Talk followed by a discussion | 25 min | Mats |
| **Relaxation techniques** | To learn and perform relaxation techniques.<br>Homework: performing relaxation techniques at home. | Demonstration and training | 15 min | Mats |
| **Close the session** | To listen to a recap of the session and be reminded of the homework.<br>To voice feelings, perceptions, and fears. | Talk followed by a discussion<br>Dialogue | 10 min | Chairs |

Session 2: Recovering from a fall.

**Table S4. Session 3: Cognitive restructuring.**

| Contents | Objetives | Method | Length | Materials Required |
|---|---|---|---|---|
| **Recap of the second session** | To explain the important concepts taught in the previous session. | Participative lesson | 10 min | Chairs, whiteboard |
| **How to act in the event of a fall** | To demonstrate how to get up in after a fall. | Demonstration and training | 10 min | Mats |
| **Cognitive restructuring** | To identify the fact that personal assertions mediate emotional reactions.<br>To become aware of dysfunctional thinking styles (polarised thinking, exaggerated generalisation, magnification, and personalisation).<br>To highlight the irrationality of certain beliefs by comparing them with reality.<br>To become aware that the impossibility of achieving desirable behaviours is often the result of irrational personal assertions.<br>To be able to identify their own misinterpretations of perceived stressors.<br>Homework: writing statements describing an alternative way of looking at the situation. | Talk followed by a discussion<br>Brainstorming<br>Talk followed by a discussion | 20 min<br>15 min<br>10 min | Computer, projector.<br>Chairs |
| **Multicomponent exercise** | To discuss the difficulties of exercising at home.<br>To demonstrate how to make a multicomponent exercise plan.<br>To give positive feedback on each individual's efforts.<br>To learn how to warn patients about the dangers of overestimating their capabilities. | Demonstration and training | 25 min | Mats |
| **Relaxation techniques** | To discuss the difficulties of performing relaxation techniques at home.<br>To demonstrate how relaxation techniques are performed.<br>To explain how to get into the habit of using relaxation techniques on a daily basis. | Demonstration and training<br>Talk followed by a discussion | 15 min | Mats |
| **Close the session** | To listen to a recap of the session and be reminded of the homework.<br>To voice feelings, perceptions, and fears. | Talk followed by a discussion.<br>Dialogue | 15 min | Chairs |

Session 3: Cognitive restructuring.

**Table S5. Session 4: Coping enhancement.**

| Contents | Objectives | Method | Length | Materials Required |
|---|---|---|---|---|
| **Recap of the third session** | To explain the important concepts taught in the previous session.<br>To share statements describing an alternative way of looking at the situation. | Participative lesson | 10 min | Chairs, whiteboard |
| **How to act in the event of a fall** | To demonstrate how to get up in after a fall. | Demonstration and training | 10 min | Mats |
| **Cognitive restructuring** | To share the irrational thoughts that were identified with the other participants. | Repertory grid | 25 min | Computer, projector |
| **Coping enhancement** | To learn how patients can identify the resources available to them to achieve their goals.<br>To learn how to help patients break down complex goals into small, manageable steps.<br>To learn how to foster relationships with people who share the same interests and objectives.<br>To explore patients' past accomplishments.<br>To be able to identify positive feedback from others. | Talk followed by a discussion<br>Brainstorming | 15 min<br>10 min | Screen Whiteboard, marker.<br>Pen, paper |
| **Multicomponent exercise** | To discuss the difficulties of exercising at home.<br>To demonstrate how to make a multicomponent exercise plan.<br>To give positive feedback on each individual's efforts.<br>To get into the habit of doing multicomponent exercise on a daily basis.<br>Homework: carrying out the exercise plan at home. | Demonstration and training<br>Talk followed by a discussion | 25 min | Mats |
| **Relaxation techniques** | To discuss the difficulties of performing relaxation techniques at home.<br>To demonstrate how relaxation techniques are performed.<br>To explain how to get into the habit of using relaxation techniques on a daily basis.<br>Homework: performing relaxation techniques at home. | Demonstration and training | 15 min | Mats |
| **Close the session** | To listen to a recap of the session.<br>To voice feelings, perceptions, and fears. | Talk followed by a discussion<br>Dialogue | 10 min | Chairs |

Session 4: Coping enhancement.

**Table S6. Session 5: Final session.**

| Contents | Objectives | Method | Length | Materials Required |
|---|---|---|---|---|
| **Recap of the fourth session** | To explain the important concepts taught in the previous session. | Participative lesson | 10 min | Chairs, whiteboard |
| **Recap of the first session** | To list risk factors for falls and fear of falling.<br>To list fall prevention strategies. | Participative lesson | 10 min | Chairs, whiteboard |
| **Recap of the second session** | To list the measures taken and their next steps. | Brainstorming | 15 min | Chairs, whiteboard |
| **How to act in the event of a fall** | To demonstrate how to get up after a fall. | Demonstration and training | 10 min | Mats |
| **Recap of cognitive restructuring** | To list irrational thoughts about fear of falling and falls. | Brainstorming | 10 min | Chairs, whiteboard |
| **Recap of coping enhancement** | To discuss positive feedback from others.<br>To share the achievements made during the workshop. | Dialogue | 20 min | Chairs |

| | | | | |
|---|---|---|---|---|
| **Multicomponent exercise** | To discuss the difficulties of exercising at home.<br>To demonstrate how to make a multicomponent exercise plan.<br>To give positive feedback on each individual's efforts.<br>To discuss the difficulties of carrying out the exercise plan at home.<br>To explain how to get into the habit of doing multicomponent exercise on a daily basis. | Demonstration and training<br>Talk followed by a discussion | 20 min | Mats |
| **Relaxation techniques** | To discuss the difficulties of performing relaxation techniques at home.<br>To demonstrate how relaxation techniques are performed.<br>To explain how to get into the habit of using relaxation techniques on a daily basis. | Demonstration and training | 10 min | Mats |
| **Close the session** | To voice feelings, perceptions, and fears experienced during the workshop.<br>To let them suggest areas for improvement.<br>To assess the feasibility and acceptability of the workshop among patients. | Dialogue<br>Assessment | 15 min | Chairs |

Session 5: Final session.

**Table S7. Session 6: Booster session.**

| Contents | Objetives | Method | Length | Materials Required |
|---|---|---|---|---|
| **Session presentation** | To get acquainted with the contents of the booster session.<br>To create a safe space. | Talk followed by a discussion | 15 min | Chairs |
| **Recap of the first session** | To list risk factors for falls and fear of falling.<br>To list fall prevention strategies. | Participative lesson | 10 min | Chairs, whiteboard |
| **How to act in the event of a fall** | To demonstrate how to get up after a fall. | Demonstration and training | 10 min | Mats |
| **Recap of recovering from a fall and fear of falling** | To list the actions taken over the last six months. | Brainstorming | 15 min | Chairs, whiteboard |
| **Recap of cognitive restructuring** | To list irrational thoughts about fear of falling and falls. | Brainstorming | 10 min | Chairs, whiteboard |
| **Recap of coping enhancement** | To share the achievements made over the last six months. | Dialogue | 15 min | Chairs |
| **Multicomponent exercise** | To demonstrate how to make a multicomponent exercise plan.<br>To give positive feedback on each individual's efforts. | Demonstration and training<br>Talk followed by a discussion | 20 min | Mats |
| **Relaxation techniques** | To demonstrate how relaxation techniques are performed.<br>To explain how to get into the habit of using relaxation techniques on a daily basis. | Demonstration and training | 10 min | Mats |
| **Close the workshop** | To voice feelings, perceptions, and fears experienced during the workshop.<br>To let them suggest areas for improvement. | Dialogue<br>Assessment | 15 min | Chairs |

Session 6: Booster session.
